# Supplementary material for: Serological Investigations on West Nile Virus in Horses in Kazakhstan
Source: Microorganisms. 2025 Nov 6;13(11):2541. doi: 10.3390/microorganisms13112541 (PMC12654397; doi:10.3390/microorganisms13112541)
Supplement: Supplementary file 1 [file microorganisms-13-02541-s001.zip › microorganisms-3919979-supplementary.pdf]

Supplementary Materials

Western blot analysis

The western blot analysis was conducted to confirm the presence of antibodies against West Nile virus (WNV) using a viral envelope antigen. All ELISA-positive sera were tested for antibodies specific to the E protein. Figure S1 shows western blot results for 6 sera from 6 regions (3 – Zhetisu, 4 – Almaty, 5 – Aktobe, 6 – Turkestan, 7 – West Kazakhstan, 8 – Atyrau). Hyperimmune mouse serum against WNV served as a positive control, while serum from healthy animals was used as a negative control.

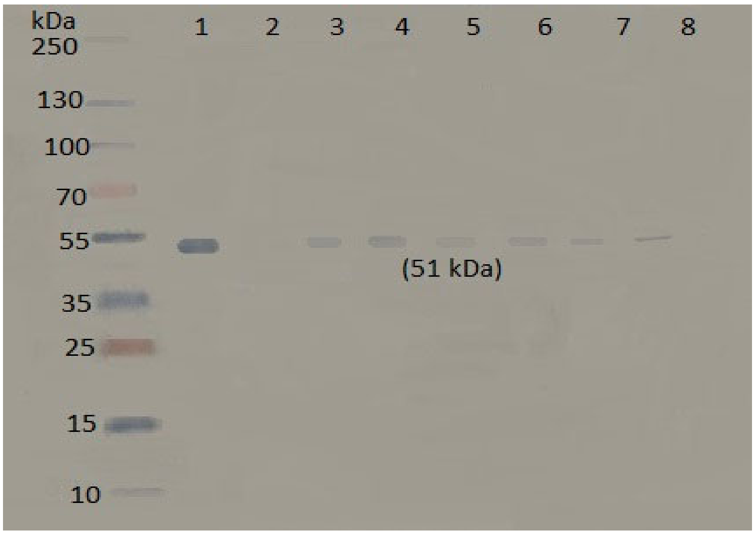

Figure S1. Western Blotting using the antigen of the West Nile fever virus and serum (diluted 1:100) from horses. Lane 1 - hyperimmune serum of mice, prepared against WNV (positive control), 2 – negative control, lanes 3–8 – horses serum (3 – Zhetisu, 4 – Almaty, 5 – Aktobe, 6 – Turkestan, 7 - West Kazakhstan, 8 – Atyrau). Marker - PageRuler Plus Prestained Protein Ladder, Thermo Scientific.

As shown in Figure S1, positive control (lane 1) demonstrated a specific reaction with the viral E protein (approximately 51 kDa), whereas the negative control (lane 2) exhibited no reactivity. During testing horse sera positive for ELISA, specific antibodies to E protein (~51 kD) were found in samples from different regions in dilution 1:100. Protein E bands were visualized in lanes 3-8, corresponding to horses from the regions of Zhetisu, Almaty, Aktobe, Turkestan, West Kazakhstan and Atyrau, respectively. Во всех остальных положительных в ИФА образцах сывороток были получены подобные результаты.

These results confirm the presence of antibodies to WNV in horses from several regions of Kazakhstan.
